# Effective Use of the Built Environment to Manage Behavioural and Psychological Symptoms of Dementia: A Systematic Review

**DOI:** 10.1371/journal.pone.0115425

**Published:** 2014-12-17

**Authors:** Lesley J. J. Soril, Laura E. Leggett, Diane L. Lorenzetti, James Silvius, Duncan Robertson, Lynne Mansell, Jayna Holroyd-Leduc, Tom W. Noseworthy, Fiona M. Clement

**Affiliations:** 1 Department Community Health Sciences, University of Calgary, Teaching Research and Wellness Building, Calgary, Alberta, Canada; 2 Institute for Public Health, University of Calgary, Teaching Research and Wellness Building, Calgary, Alberta, Canada; 3 Institute of Health Economics, Edmonton, Alberta, Canada; 4 Department of Medicine, University of Calgary, Foothills Medical Centre South Tower, Calgary, Alberta, Canada; 5 Alberta Health Services, Calgary, Alberta, Canada; Federal University of Rio de Janeiro, Brazil

## Abstract

**Objective:**

To determine the effectiveness of built environment interventions in managing behavioural and psychological symptoms of dementia (BPSD) among residents in long-term care settings.

**Methods:**

Systematic review of literature published from 1995–2013. Studies were included if they: were randomized controlled trials, quasi-experimental trials, or comparative cohort studies; were in long-term or specialized dementia care; included residents with dementia and BPSD; and examined effectiveness of a built environment intervention on frequency and/or severity of BPSD. Quality of included studies was assessed using the Downs and Black Checklist. Study design, patient population, intervention, and outcomes were extracted and narratively synthesized.

**Results:**

Five low to moderate quality studies were included. Three categories of interventions were identified: change/redesign of existing physical space, addition of physical objects to environment, and type of living environment. One of the two studies that examined change/redesign of physical spaces reported improvements in BPSD. The addition of physical objects to an existing environment (n = 1) resulted in no difference in BPSD between treatment and control groups. The two studies that examined relocation to a novel living environment reported decreased or no difference in the severity and/or frequency of BPSD post-intervention. No studies reported worsening of BPSD following a built environment intervention.

**Conclusions:**

The range of built environment interventions is broad, as is the complex and multi-dimensional nature of BPSD. There is inconclusive evidence to suggest a built environment intervention which is clinically superior in long-term care settings. Further high-quality methodological and experimental studies are required to demonstrate the feasibility and effectiveness of such interventions.

## Introduction

Alzheimer's disease and related dementias are chronic, progressive disorders that result in the impairment of cognitive functions, including memory, orientation, comprehension, and executive function [1]–[3]. The debilitating and disorientating nature of dementia, coupled with the older age of the prevalent population, results in a substantial proportion of individuals with dementia becoming institutionalized in long-term care (LTC) facilities [4]–[6]. In addition to the impact of cognitive impairment, the accompanying responsive behaviours that may occur [7], also known as behavioural and psychological symptoms of dementia (BPSD), add additional challenges for patients, families, caregivers and staff of LTC facilities [1], [2].

Commonly described as a heterogeneous set of complex symptoms manifesting as agitation, disinhibition, physical and/or verbal aggression, anxiety, depression and delusions, BPSD requires a multifaceted approach to achieve successful management [5]. Treatment options for managing BPSD have typically involved pharmacological approaches, including the use of antipsychotics, cholinesterase inhibitors and antidepressants [8], [9]. Recent systematic reviews concerning the use of pharmacological interventions for the treatment of BPSD, particularly antipsychotic medications, conclude that while these drugs may be moderately effective or ineffective at reducing the frequency and/or severity of responsive behaviours, they are associated with an increased risk of major adverse events (e.g., stroke) and death [10], [11]. In contrast, there is growing evidence to suggest that the management of BPSD in LTC should shift from the traditional practice of medication-based symptom management, to comprehensive non-pharmacological approaches grounded on maintaining the physical and emotional comfort of the individual within their environment [8], [11], [12]. Such non-pharmacological interventions may be applied to an individual (e.g. massage, music therapy and animal-assisted therapy) [13], [14] or related to the physical living setting or built environment [11], [12], [15].

Previous studies examining modifications to the built environment have drawn from a number of design principles and frameworks for dementia care homes and suggest that purposeful design of one's surroundings may play an active role in promoting a sense of well-being and improved functionality [16]–[20] While no singular definition of the built environment has been universally adopted [21], it is commonly understood as the constructed, physical surroundings (interior and exterior) where an individual conducts activities of daily living such as eating, bathing and sleeping, and interacts socially [19]. Therefore an intervention to the built environment would constitute any direct manipulation of the physical structure where an individual resides, be it their personal residence or a shared LTC facility [17], [22]. Specific examples of such interventions have included esthetic redesign or addition of new objects to specific rooms, construction of indoor and outdoor areas in existing residences or facilities, and even relocation of individuals to a completely novel living environment [21].

Despite the recent advances in the breadth and depth of built environment interventions, their effectiveness in managing BPSD specifically within LTC settings remains unclear. The objective of this systematic review is to determine the effectiveness of built environment interventions, in comparison to usual care or no intervention, on the frequency and/or severity of BPSD among residents in LTC.

## Materials and Methods

A systematic review was completed in accordance with the Preferred Reporting Items for Systematic Reviews and Meta-Analyses (PRIMSA; see S1 Checklist) [23]. No protocol exists for this systematic review. As this research did not involve participation of human subjects, research ethics board review was not required, nor was informed consent.

### Selection of the Literature

The search strategy was developed with an information specialist. An inclusive search of MEDLINE, CENTRAL Register of Controlled Trials, EMBASE, PsycINFO, Cochrane Database of Systematic Reviews, HTA Database, NHSEED, Environment Complete, Social Work Abstracts, SocINDEX, CINAHL, Urban Studies Sociological abstracts, and Social Services Abstracts was performed from 1995 to June 2013. The search strategy focused on combining terms for dementia, such as “dementia”, “Alzheimer's disease”, and “Alzheimer's”, and built environment interventions, such as “environment design”, “facility design and construction”, “hospital design and construction”, and “health facility environment.” Results were filtered to exclude non-human studies and languages other than English or French; no other limits were used. The search strategies for all electronic databases are outlined in S1 Appendix.

Articles that reported original data, were designed as randomized controlled trials (RCT), quasi-experimental trials, or prospective comparative cohort studies, were set in a long-term care (LTC) facility or a unit or facility specializing in dementia care, included residents with dementia diagnosed with behavioural and psychological symptoms in dementia (BPSD), involved an environmental intervention specific to the physical or built structure of the living environment (e.g. architectural design, building reconstruction, interaction/use of the physical environment by staff or residents) and reported outcomes related to the clinical efficacy of built environment interventions on BPSD were selected (S2 Appendix). Reference lists of identified articles were also hand-searched for additional relevant articles not captured in the search. Abstract and full-text review of articles was completed in duplicate. All abstracts selected for inclusion by either reviewer proceeded to full-text review. This initial screen was conducted using broad criteria to ensure that all relevant literature was captured. During full-text review, any disagreement between reviewers was resolved through discussion and consensus. A kappa statistic for reviewer agreement was calculated.

### Data Extraction and Synthesis

Data from the included studies was extracted in duplicate using a standard data extraction form. Any discrepancy was resolved through discussion and consensus. Data related to study design, publication date, country, number of participants, dementia diagnosis, healthcare setting, procedure or intervention information, outcome measures and relevant results reported by each study were extracted and narratively synthesized. Specifically, reported outcome measures included decreases in frequency and/or severity of responsive behaviours or episodes of BPSD. Heterogeneity in reported outcomes and/or summary measures precluded pooling of data for meta-analysis.

### Quality Assessment

Each included study was assessed for quality using the Downs and Black Checklist [24]. This checklist includes 27 criteria, widely covering areas reporting quality, external and internal validity, and power. The quality of each study was independently assessed by two reviewers, with discrepancies resolved through discussion and consensus.

## Results

### Study Selection

**Figure 1:**
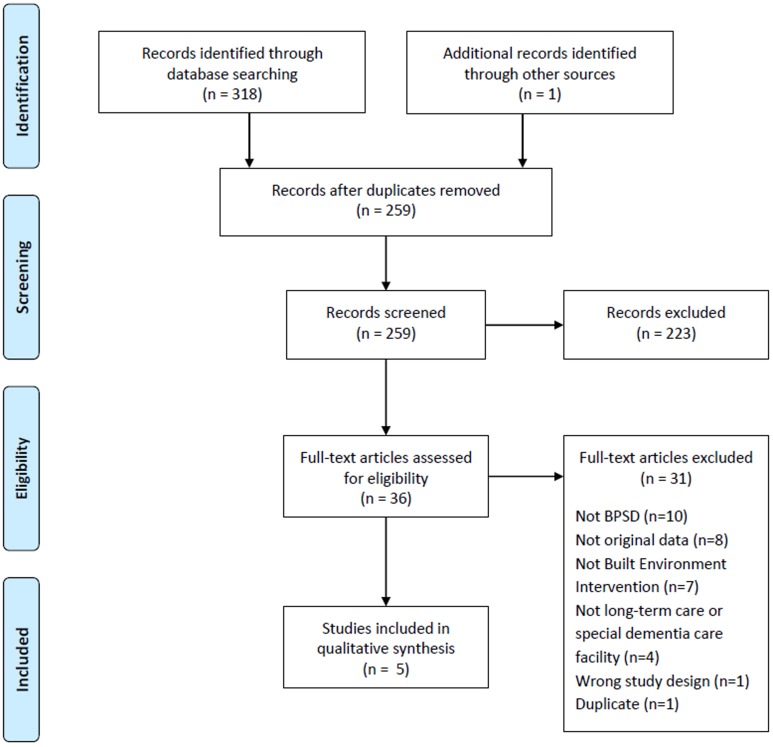
PRISMA Flow Diagram of Included Studies. A total of 318 abstracts were identified from the literature search. Hand-searching of articles included in previously published systematic reviews identified 1 relevant article. After removal of duplicate records, 259 abstracts were reviewed and 223 were excluded. Thirty-six articles were assessed in full-text, 31 of which were excluded and, ultimately, five comparative cohort studies were included in the final qualitative synthesis. Due to heterogeneity in reported outcomes and/or summary measures, pooling of data for meta-analysis was not possible.

The literature search identified 258 unique abstracts. Previous high-quality systematic reviews were hand-searched for articles that met the inclusion criteria and 1 relevant article was identified. Two-hundred and twenty-three citations were excluded in the abstract review and 36 were assessed in full-text. Thirty-one articles were excluded following full-text review (Kappa  = 0.52, 95% CI 0.21–0.83) and ultimately, five studies were included in the final analysis (Fig. 1).

### Quality of Included Studies

The included studies were of low to moderate quality. Using the Downs and Black Checklist, the included studies scored between 14 [25], [26] and 18 [27] points for quality, out of a possible 28 points (Table 1). All of the studies clearly described the intervention, representativeness of facility, staff and residents, the analysis, resident compliance and assessment of outcome measures. None of the included studies provided characteristics of the total population or the sample population, thereby, limiting assessment of the representativeness of the sample. Due to the study designs and nature of the interventions, randomization and blinding were not possible.

**Table 1:** Downs and Black Checklist for Quality Assessment.

		Cox (2004) [25]	Edgerton (2010) [26]	Namazi (1992) [28]	Reimer (2004) [15]	Wilkes (2005) [27]
**REPORTING**						
**Q1**	**Hypothesis/aim/objective clearly described**	1	0	1	1	1
**Q2**	**Main outcomes in Introduction or Methods**	1	0	1	1	1
**Q3**	**Patient characteristics clearly described**	1	1	1	1	1
**Q4**	**Interventions of interest clearly described**	1	1	1	1	1
**Q5**	**Principal confounders clearly described**	0	0	0	0	0
**Q6**	**Main findings clearly described**	1	1	1	1	1
**Q7**	**Estimates of random variability provided for main outcomes**	0	0	0	0	0
**Q8**	**All adverse events of intervention reported**	0	1	1	0	0
**Q9**	**Characteristics of patients lost to follow-up described**	0	1	0	0	1
**Q10**	**Probability values reported for main outcomes**	0	0	0	0	0
**EXTERNAL VALIDITY**						
**Q11**	**Subjects asked to participate were representative of source population**	UTD	UTD	UTD	UTD	UTD
**Q12**	**Subjects prepared to participate were representative of source population**	UTD	UTD	UTD	UTD	UTD
**Q13**	**Location and delivery of study treatment was representative of source population**	1	1	1	1	1
**INTERNAL VALIDITY – BIAS & CONFOUNDING**						
**Q14**	**Study participants blinded to treatment**	0	0	0	0	0
**Q15**	**Blinded outcome assessment**	0	0	0	0	0
**Q16**	**Any data dredging clearly described**	1	1	1	1	1
**Q17**	**Analyses adjust for differing lengths of follow-up**	1	1	1	1	1
**Q18**	**Appropriate statistical tests performed**	1	0	0	1	0
**Q19**	**Compliance with interventions was reliable**	1	1	1	1	1
**Q20**	**Outcome measures were reliable and valid**	1	1	1	1	1
**Q21**	**All participants recruited from the same source population**	1	1	1	1	1
**Q22**	**All participants recruited over the same time period**	1	1	1	1	1
**Q23**	**Participants randomized to treatment(s)**	0	0	0	0	0
**Q24**	**Allocation of treatment concealed from investigators and participants**	0	0	0	0	0
**Q25**	**Adequate adjustment for confounding**	0	1	1	1	1
**Q26**	**Losses to follow-up taken into account**	UTD	UTD	UTD	UTD	1
**POWER**						
**Q27**	**Sufficient power to detect treatment effect at significance level of 0.05**	1	0	1	1	1
**TOTAL**		**14**	**14**	**15**	**17**	**18**

1 UTD: Unable to determine.

### Characteristics of Included Studies

All 5 included studies were non-randomized comparative cohort studies published between 1992 and 2010. Four of the included studies were pre- and post-intervention design. The control group from the remaining study was sampled from two separate LTC facilities compared to those in the intervention group [15]. Characteristics from each of these studies are summarized in Table 2. Two studies were conducted in Australia [25], [27]; and the remaining 3 studies were conducted in Canada [15], Scotland [26], and the United States [28]. The majority of the included studies (n = 4) were conducted in designated LTC facilities [15], [25]–[27] and 1 study conducted in a single unit or facility that specialized in dementia care [28]. The sample size varied across studies, between 16 [27] and 185 [15] participants per study.

**Table 2:** Characteristics and Results of Included Studies.

Author (Year)	Country	Dementia Diagnosis	Health Care Setting	Intervention Group (N)	Control Group (N)	Intervention Details	Dose/Intensity	Outcome Measure(s)	Main Results
**Change or Redesign of Existing Physical Structures or Spaces**									
Edgerton [26] (2010)	Scotland	Unspecified dementia	One specialized long term care facility	47	53	Redesign of an existing corridor	Three months of living with redesigned corridor	Resident behaviour	No significant difference in overall resident behaviour while in corridor.
Namazi [28] (1992)	United States	Probable Alzheimer's disease	One special care facility	22	22	Unlocked doors to courtyard	Five hours a day over 10 days – total of 50 hours per condition	Agitation and behaviour	Decreased agitation when doors were unlocked compared to when doors were locked.
**Addition of Physical Objects or Spaces to the Existing Environment**									
Cox [25] (2004)	Australia	Unspecified “dementia”	One nursing home	24	24	Two multisensory environments - landscaped garden and Snoezelen room	Three 16 minute sessions in control, Snoezelen room and garden –total of nine 16 minute sessions	Affect: pleasure; anger; anxiety/fear; sadness; interest; contentment	No significant differences in affect states before and after intervention, between the 3 environments. The only significant difference was that more sadness was recorded in the living room environment compared to the garden or Snoezelen room.
**Relocation to a Novel Living Environment**									
Reimer [15] (2004)	Canada	Unspecified “dementia”	Twenty four long-term care facilities with four designated assisted living environments	62	123	Move to purpose-built specialized care facilities	One year living in specialized care facility	Quality of Life, including: agitation; activities of daily living; interest in environment; social withdrawal; depression; concentration; memory	Quality of life in intervention group was similar or better compared to control group.
Wilkes [27] (2005)	Australia	Unspecified “dementia”	One long-term care facility	16	16	Move to a Special Care Unit	Six months living in either specialized care unit or regular unit	Agitation behaviour, including: aggressive, physically; non-aggressive, verbal	Reduction of verbally agitated behaviour in intervention group compared to control group.

Three general categories of intervention were identified: a change or redesign of existing physical structures or spaces within the environment [26], [28]; the addition of physical objects or spaces to the existing environment [25]; and the relocation of the study population to a novel living environment [15], [27].

### Change or Redesign of Existing Physical Structures or Spaces

Two of the included studies examined the impact of changing or redesigning the existing physical structures or spaces within the living environment on the behaviours of study participants [26], [28]. In a pre- and post-intervention study conducted by Namazi *et al.* (1992) the basic intervention of unlocking doors to the common courtyard of a specialized care facility reportedly led to decreased agitation amongst residents with dementia compared to the control conditions when the doors were locked [28]. In contrast, one study that examined a large-scale redesign of an existing main corridor of a large psychiatric hospital found that there was no difference in the measured resident behaviours before and 3-months after the built environment intervention [26].

### Addition of Physical Objects or Spaces to the Existing Environment

One study examined the impact of adding new physical objects or features to an existing component of the built environment. Specifically, Cox *et al.* (2004) measured the level of affect in participating residents with dementia during time spent in the control living room setting compared in two multisensory environments added to the LTC facility: a Snoezelen room and a landscaped garden [25]. There were no differences observed in the various affect states before and after time spent in the 3 environments; the only significant difference was the recording of more ‘sadness’ in the living room relative to either multisensory environment [25].

### Relocation to a Novel Living Environment

Two studies examined the impact of several responsive or agitated behaviours following a complete relocation in living environment. Relocation of a small sample of 16 residents from a traditional care unit to a specialized dementia care unit in a single LTC facility resulted in decreased agitation amongst participants, specifically in verbally aggressive behaviour, 6-months post-intervention [27]. In a larger study conducted by Reimer *et al.* (2004), of 185 residents with dementia, the quality of life, which included measures of responsive behaviours such as agitation, socially appropriate behaviour, social withdrawal and interest in the environment, were compared between participants in traditional LTC facilities (control group) and in a purpose-built specialized dementia care facility [15]. One year post-intervention, the authors found that there was greater sustained interest in the environment, less negative affect, and the overall quality of life was similar or better for the intervention group compared to controls; however, there were no significant differences with regards to concentration, orientation, socially appropriate behaviour, and social withdrawal between study groups.

## Discussion

Five relevant studies were identified in the present systematic review of built environment interventions for the management of BPSD in LTC. The included studies were all designed as comparative cohort studies, had relatively small sample sizes, and were of low to moderate quality. With no standardized system to classify interventions to the built environment [21], we adopted an inductive approach to thematically organize the identified interventions into 3 general categories: a change or redesign of existing physical structures or spaces within the environment [26], [28]; the addition of physical objects or spaces to the existing environment [25]; and the relocation of the study population to a novel living environment [15], [27]. Overall, two of the five included studies demonstrated improvements and 3 reported no difference in the frequency and/or severity of BPSD following the built environment intervention. No studies reported worsening of BPSD following an intervention.

Manipulations to the built environment and other non-pharmaceutical approaches have been widely discussed as first-line therapies to managing BPSD in institutionalized seniors [12]. The present review provides little conclusive evidence to suggest superior effectiveness of any single, built environment intervention. However, this does not invalidate the use of built environment interventions or their potential synergistic effectiveness when combined with other non-pharmacological approaches, such as sensory therapy, recreational therapy, and/or social contact [29]. In fact, given the number of guidelines and consensus statements that recommend the use of combinatorial non-pharmacological approaches for managing BPSD [8], [13], [14], manipulations to the built environment, alone, are not likely to address the spectrum of responsive behaviours observed in LTC settings.

The variability in manipulations to the built environment observed amongst studies demonstrates great creative capacity with regards to these interventions. However, with the limited number of included studies, the small numbers of participants, and given that most of the included studies were from single sites it is difficult to predict the feasibility for large-scale implementation of such interventions at the health system level. Inadequate reporting of the required resources and costs across studies also precluded inferences of the most cost-effective of interventions; though it is apparent that some are likely to be more costly and burdensome relative to others. For example, relocating an entire building of LTC residents to a new purposively designed LTC facility requires substantially different resources compared to the unlocking of doors in an existing building. Thus, decisions to undertake a given built environment intervention must balance the specific needs of the residents and care-givers/staff as well as the appropriateness of the intervention, with the inherent cost and resource limitations of the facility or health system.

The findings of this present study are consistent with those reported by previous systematic reviews. The systematic review conducted by Gitlin *et al*. (2003) examined broader categories of environmental interventions beyond the physical construct of one's living space (e.g. sensory and olfactory therapies, tasks and activities performed in the environment, and a combination of these interventions) and similarly noted variability in intervention protocols, sample sizes, quality of evidence and extent of outcome data reported [21]. Such variability among interventions can be seen as both a strength (i.e. demonstrates the range of creative and feasible manipulations), and a weakness (i.e. makes it difficult to draw conclusions from the evidence base) [21]. Other past evidence syntheses were also restricted to thematic analysis and narration of studies by inductive intervention categories [17], [21], [30] Further high-quality methodological and experimental research is needed in this area to help guide the facilities responsible for caring for persons with dementia and BPSD.

There are several limitations to the synthesis of the evidence in this review. By focussing specifically on interventions to the physical or built structure of the living environment for residents with BPSD in LTC or specialized dementia care units or facilities, other interventions applied to the built environments of an individual's own home, temporary or transition housing, seniors' daycare facilities, or acute care facilities were excluded from this review. Interventions that involved manipulations experienced by the residents within the environment, but without any physical or structural manipulation to the environment (e.g. music therapy) were also excluded. Most studies also had short-term follow-up periods (no longer than a few months post-intervention), rendering it difficult to determine whether results can be sustained or generalized over the long-term. Lastly, as mentioned, heterogeneity within intervention categories and insufficiency in reported outcome data, as well as the overall low quality of the included studies precluded meta-analyses.

## Conclusions

The complex and multi-dimensional nature of BPSD requires a multifaceted management approach [5]. Responsiveness to an intervention is likely to be highly individualized, with the degree of response to therapy based on an individual's background and the complexity of their symptoms. The interventions to the built environment examined within this present review serve as a reminder that one's physical and social surroundings have large influence over one's psychological well-being. However, there remains a dearth of high-quality evidence to conclusively guide the selection of any particular built environment intervention. Given the growing evidence concerning the effectiveness of other non-pharmacological approaches to managing BPSD, changes to the built environment likely serve as only one component of the arsenal of therapies in managing BPSD among residents in LTC.

## Supporting Information

S1 Checklist **PRISMA 2009 Checklist.**
(DOC)

S1 Appendix **Search Strategies for the Literature Search (1995–June 2013).**
(DOCX)

S2 Appendix **Inclusion and Exclusion Criteria for the Systematic Review.**
(DOCX)
