# Supplementary material for: Effective Use of the Built Environment to Manage Behavioural and Psychological Symptoms of Dementia: A Systematic Review
Source: PLoS One. 2014 Dec 17;9(12):e115425. doi: 10.1371/journal.pone.0115425 (PMC4269426; doi:10.1371/journal.pone.0115425)
Supplement: S1 Appendix — Search Strategies for the Literature Search (1995–June 2013). (DOCX) [file pone.0115425.s002.docx]

**APPENDIX S2.** Search Strategies for the Literature Search (1995-June 2013)

**MEDLINE (OVID)**

**Cochrane CENTRAL Register of Controlled Trials (OVID)**

1. exp Dementia/

2. exp Alzheimer Disease/

3. (dementia or alzheimer*).tw.

4. 1 or 2 or 3

5. built environment*.tw.

6. Environment Design/

7. "facility design and construction"/ or "hospital design and construction"/

8. health facility environment/

9. 5 or 6 or 7 or 8

10. 4 and 9

11. limit 10 to (yr="1995 -Current" and (english or french))

12. limit 11 to animals

13. limit 11 to (animals and humans)

14. 12 not 13

15. 11 not 14

**EMBASE (OVID)**

1. exp *dementia/

2. exp *Alzheimer disease/

3. (dementia or alzheimer*).tw.

4. 1 or 2 or 3

5. environment/

6. hospital design/

7. built environment*.tw.

8. built environment.mp.

9. (built environment or built environment assessment or built environmental change or built environmental pattern or built infrastructure).sh.

10. 5 or 6 or 7 or 8 or 9

11. 4 and 10

12. limit 11 to ((english or french) and yr="1995 -Current")

13. limit 12 to animals

14. limit 12 to (human and animals)

15. 13 not 14

16. 12 not 15

17. limit 16 to (book or book series or editorial or letter)

18. 16 not 17

19. limit 18 to conference abstract

20. 7 and 19

21. 18 not 19

22. 20 or 21

**PsycINFO (OVID)**

1. exp Dementia/

2. exp Alzheimer's Disease/

3. (dementia or alzheimer*).tw.

4. 1 or 2 or 3

5. therapeutic environment/ or exp facility environment/

6. exp Built Environment/

7. built environment*.tw.

8. 5 or 6 or 7

9. 4 and 8

10. exp Environmental Planning/

11. 4 and 10

12. 9 or 11

13. limit 12 to ((english or french) and yr="1995 -Current")

**Cochrane Database of Systematic Reviews**

**HTA Database (OVID)**

**NHSEED (OVID)**

1. (environment* or design* or facility planning).tw.

2. (dementia or alzheimer*).tw.

3. 1 and 2

4. limit 3 to ((english or french) and yr="1995 -Current")

**Environment Complete (EBSCO)**

**Social Work Abstracts (EBSCO)**

**SocINDEX (EBSCO)**

**CINAHL (EBSCO)**

**Urban Studies (EBSCO)**

1. (environment* or design* or facility planning)[All Fields]

2. (dementia or alzheimer*)[All Fields]

3. 1 and 2

4. limit 3 to ((english or french) and yr="1995 -Current")

**Sociological abstracts (ProQuest)**

**Social Services Abstracts (ProQuest)**

1. (environment* or design* or facility planning)[Anywhere]

2. (dementia or alzheimer*)[Anywhere]

3. 1 and 2

4. limit 3 to ((english or french) and yr="1995 -Current")
