# Supplementary material for: Effective Use of the Built Environment to Manage Behavioural and Psychological Symptoms of Dementia: A Systematic Review
Source: PLoS One. 2014 Dec 17;9(12):e115425. doi: 10.1371/journal.pone.0115425 (PMC4269426; doi:10.1371/journal.pone.0115425)
Supplement: S2 Appendix — Inclusion and Exclusion Criteria for the Systematic Review. (DOCX) [file pone.0115425.s003.docx]

**APPENDIX S3.** Inclusion and Exclusion Criteria for the Systematic Review

| **Inclusion Criteria** | **Exclusion Criteria** |
| --- | --- |
| Behavioural and psychological symptoms of dementia (BPSD) or responsive behaviours in dementia  Long-term care (LTC) or unit or facility specialized in dementia care  Environmental interventions (e.g. architectural design, decorative change, relocation in physical space, etc.)  Outcome measure related to BPSD (change in frequency and/or severity)  Original Data  Randomized or Nonrandomized Quasi-experimental Trials  Prospective Comparative Cohort Studies  English or French language | No BPSD, no dementia  Pediatric population  Other non-pharmacological treatments, not focused on the built environment  Not in LTC or a special dementia care facility  Non-original data  Grey Literature  Not RCTs or Comparative Cohort Studies  Preclinical/Animal studies  Not English or French language |
